# Potential role of oxidative exoenzymes of the extremophilic fungus *Pestalotiopsis palmarum* BM-04 in biotransformation of extra-heavy crude oil

**DOI:** 10.1111/1751-7915.12067

**Published:** 2013-07-01

**Authors:** Leopoldo Naranjo-Briceño, Beatriz Pernía, Mayamaru Guerra, Jhonny R Demey, Ángela De Sisto, Ysvic Inojosa, Meralys González, Emidio Fusella, Miguel Freites, Francisco Yegres

**Affiliations:** 1 Área de Energía y Ambiente, Instituto de Estudios Avanzados (IDEA)Sartenejas, Caracas, 1080, Venezuela; 2 Unidad de Optoelectrónica y Tecnología Láser, Instituto Zuliano de Investigaciones Tecnológicas (INZIT)Maracaibo, Estado Zulia, Venezuela; 3 Laboratorio de Investigación y Apoyo Docente del Santa Ana (LIADSA), Universidad Nacional Experimental Francisco de Miranda (UNEFM)Estado Falcón, Venezuela

## Abstract

Large amount of drilling waste associated with the expansion of the Orinoco Oil Belt (OOB), the biggest proven reserve of extra-heavy crude oil (EHCO) worldwide, is usually impregnated with EHCO and highly salinized water-based drilling fluids. Oxidative exoenzymes (OE) of the lignin-degrading enzyme system (LDS) of fungi catalyse the oxidation of a wide range of toxic pollutants. However, very little evidences on fungal degradation or biotransformation of EHCO have been reported, which contain high amounts of asphaltenes and its biodegradation rate is very limited. The aims of this work were to study the ability of *Pestalotiopsis palmarum* BM-04 to synthesize OE, its potential to biotransform EHCO and to survive in extreme environmental conditions. Enzymatic studies of the LDS showed the ability of this fungus to overproduce high amounts of laccase (LACp) in presence of wheat bran or lignin peroxidase (LIPp) with EHCO as sole carbon and energy source (1300 U mgP^−1^ in both cases). FT-IR spectroscopy with Attenuated Total Reflectance (ATR) analysis showed the enzymatic oxidation of carbon and sulfur atoms in both maltenes and asphaltenes fractions of biotreated EHCO catalysed by cell-free laccase-enriched OE using wheat bran as inducer. UV-visible spectrophotometry analysis revealed the oxidation of the petroporphyrins in the asphaltenes fraction of biotreated EHCO. Tolerance assays showed the ability of this fungus to grow up to 50 000 p.p.m. of EHCO and 2000 mM of NaCl. These results suggest that *P. palmarum* BM-04 is a hopeful alternative to be used in remediation processes in extreme environmental conditions of salinity and EHCO contamination, such as the drilling waste from the OOB.

## Introduction

The massive development of the Orinoco Oil Belt (OOB), the biggest proven reserve of EHCO worldwide located in the Bolivarian Republic of Venezuela, generates a large amount of drilling waste, commonly impregnated with EHCO and highly salinized and corrosive water-based drilling fluids used in the oil drilling processes, which should be properly managed to ensure non-contamination of soil and water, complying with ethics and environmental standards (León *et al*., 2009). The biotechnology applied to the transformation of oil pollutants such as drilling waste to lead a reduction or elimination of their environmental impact is subject of study in our research group at the Instituto de Estudios Avanzados (IDEA) in the Bolivarian Republic of Venezuela.

The crude oil is a heterogeneous mixture of organic compounds, mainly water-insoluble hydrocarbons, which can be separated into four major fractions called SARA (Saturates, Aromatics, Resins and Asphaltenes). The mixture that compresses SAR fractions is referred to the maltenes. Asphaltenes are a heterogeneous and complex mixture formed by high-molecular-weight compounds not soluble in n-heptane or n-pentane, and soluble in benzene or toluene, which contain heteroatoms (S, O and N) and heavy metals such as Ni and V in their structure (Fish *et al*., 1984; Waldo *et al*., 1991; Strauz *et al*., 1992; Uribe-Álvarez *et al*., 2011; Ayala *et al*., 2012). Furthermore, asphaltenes are recognized as the heaviest and most polar fraction of crude oil, concomitant most recalcitrant and less available to be degraded by microorganisms. Its recalcitrancy may be explained by its high degree of aromaticity combined by the presence of short alkyl chains (Ayala *et al*., 2012).

Ligninolytic fungi are the main organisms involved in the carbon recycling from lignin. In addition, they are able to transform a diversity of substrates, including pollutants and toxic compounds (Dávila and Vázquez-Duhalt, 2006).

Fungal mineralization or transformations of EHCO and their maltenes or asphaltenes fractions is very little reported and controversial. Oudot and colleagues (1993), described the ability of the fungi *Emericella nidulans, Graphium putredinis, Eupenicillium javanicum* and *Aspergillus flavipes* to degrade resins and asphaltenes in a range of 15–28% and 15–40% respectively. Chaillan and colleagues (2004), reported the ability of filamentous fungi *Paecilomyces variotii* and *Fusarium decemcellulare*, and the yeasts *Candida palmioleophila* and *Pichia guillermomdii* to degrade a range of 10–15% of resins and asphaltenes. Fedorak and colleagues (1993) described the transformations of petroporphyrins and asphaltenes by chloroperoxidase (CPO) of *Caldariomyces fumago*, a protein with high peroxidase activity and versatility that is able to halogenate aromatic molecules like polycyclic aromatic hydrocarbons (PAHs); unfortunately, it has been reported as a feeble protein (García-Arellano *et al*., 2004; Uribe-Álvarez *et al*., 2011). Later, García-Arellano and colleagues (2004) reported that the chemically modified Cytochrome C catalysed the oxidation of sulfur and carbon atoms in the petroporphyrin rich-fraction of asphaltenes, leading the removal of 74% and 95% of Ni and V respectively. More recently, Ayala and colleagues (2012) described the biotransformation of porphyrin-free asphaltenes fraction catalysed by CPO-based biocatalyst in order to reduce coke formation during thermal decomposition in the oil industry. Uribe-Álvarez and colleagues (2011) described the ability of a strain of *Neosartorya fischeri*, isolated from Guanoco natural asphalt lake of Sucre State, Venezuela, to grow using asphaltenes as sole carbon and energy source, and to mineralize the 13.2% of asphaltenes. Interestingly, these authors reported that the laccase activity was only detected in the medium with asphaltenes as sole carbon and energy source. Laccases are multicopper-containing enzymes widely distributed in higher plants and fungi that catalyse the oxidation of phenolic and non-phenolic compounds with the concurrent reduction of molecular oxygen to water (Otterbein *et al*., 2000). The laccase is a versatile enzyme for multiple biotechnological applications, including the enzymatic bleaching of kraft pulp, delignification, polluted-soils bioremediation and oxidation of PAHs (Kunamneni *et al*., 2007).

*Pestalotiopsis* genus is the most commonly isolated endophytic fungi of tropical plants and has been shown to produce a wide range of bioactive secondary metabolites (Maharachchikumbura *et al*., 2011; Yang *et al*., 2012), to decolorize synthetic dyes (Hao *et al*., 2006), to efficiently decompose lignocelluloses substrates (Saparrat and Hammer, 2006), to biodegrade synthetic polymer polyester polyurethane (Russell *et al*., 2011), and to overproduce laccase in solid-state fermentation of lignocellulosic by-products as substrates (Chen *et al*., 2011).

Nevertheless, the role of *Pestalotiopsis* as oil-degrading fungus has been little studied. In fact, the sole evidence of a strain of *Pestalotiopsis* able to use several PAHs (pyrene, phenanthrene, dibenzothiophene and, naphthalene) as carbon and energy source has been reported by Naranjo and colleagues (2007). Husaini and colleagues (2008) and Kim and colleagues (2010) described the isolation of *Pestalotiopsis* strains from a soil contaminated with motor oil and from creosote-treated wood respectively.

The goals of this work were to determine: (i) the ability of *Pestalotiopsis palmarum* BM-04 to synthesize OE of the LDS in the presence of lignocellulolytic substrate (wheat bran) or EHCO as sole carbon and energy sources, (ii) the potential of the cell-free laccase-enriched OE from *P. palmarum* BM-04 to catalyse the biotransformation of EHCO, and (iii) the tolerance of *P. palmarum* BM-04 to grow in extreme conditions such as high concentration of salinity and EHCO, to be used as fungal biocatalyst in the mycoremediation processes of oil-polluted soils. Also, the BM-04 strain is newly identified according to standards approved by the Consortium for the Barcode of Life.

## Results

### Molecular and taxonomic identification of *P. palmarum* BM-04

*Pestalotiopsis palmarum* BM-04 was previously identified through PCR amplification and sequencing of the 28S rRNA gene (Naranjo *et al*., 2007). However, the internal transcribed spacer (ITS) region was recently approved as a barcode for fungi by the Consortium for the Barcode of Life (http://www.barcoding.si.edu/). For this reason, a new molecular identification was performed using ITS region that comprises 18S ribosomal RNA, internal transcribed spacer 1, 5.8S ribosomal RNA, internal transcribed spacer 2 and 28S ribosomal RNA gene. DNA sequencing results showed 100% of identity (GenBank Accession No. JX492321) to *Pestalotiopsis* aff. *palmarum* PP119 (GenBank Accession No. FJ884135). This result was confirmed by taxonomical characterization studies (data not shown). Molecular and taxonomical studies combined with the previously reported by Naranjo and colleagues (2007), allow us to determine that BM-04 strain belongs to *P. palmarum*.

### *P. palmarum* BM-04 is able to overproduce LACp with wheat bran or LIPp with EHCO when are used as sole carbon and energy source respectively

**Figure 1:**
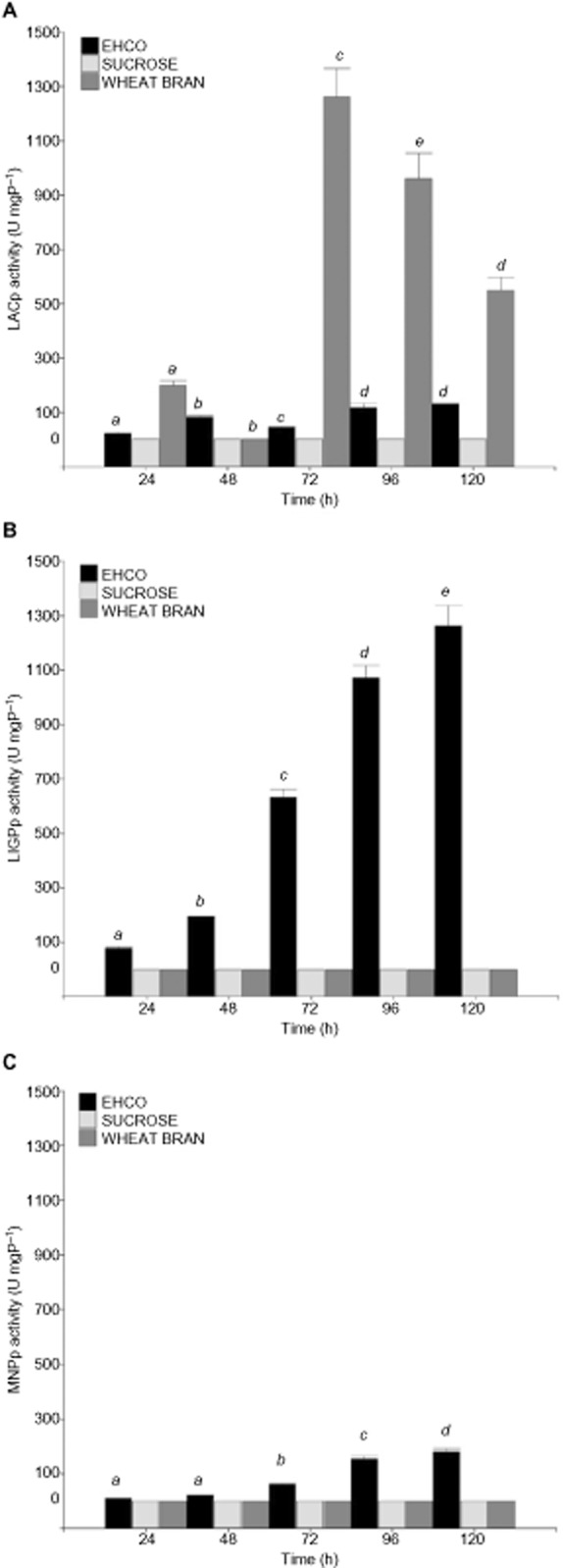
Average of specific OE activities of the LDS in Cx medium with EHCO, wheat bran or sucrose (control) as sole carbon and energy source at 24, 48, 72, 96 and 120 h of growth: (A) LACp, (B) LIPp and (C) MNPp. Dissimilar letters show significant differences (*P* ≤ 0.05). LACp, laccase; LIPp, lignin peroxidase; MNPp, manganese peroxidase.

In order to test the ability of *P. palmarum* BM-04 to synthesize OE of the LDS, the strain was grown in liquid minimal medium supplemented with wheat bran (lignocellulolytic substrate) or EHCO as sole carbon and energy sources; and LACp, LIPp and manganese peroxidase (MNPp) activities were measured. Sucrose carbon source was used as control. The DGC multiple comparison test was used to detected significant differences among treatment and control. The results showed (Fig. 1A–C) that the studied activities were not induced with sucrose. LACp activity (Fig. 1A) was strongly induced with wheat bran at 72 h and then decreased to 96 and 120 h. In this condition, LACp activity was increased 1379-, 931- and 1209-fold higher at 72, 96 and 120 h with regard to the control (1327.38; 987.87 and 561.79 U mgP^**−**1^ respectively), obtaining significant differences (*P* ≥ 0.05). In presence of EHCO, LACp activity was lower induced but even with significant differences with regard to the control (*P* ≥ 0.05). The LIPp activity (Fig. 1B) was only, strongly and linearly induced with EHCO as sole carbon source. In this condition, LIPp activity was increased 1350-, 1012-, 2122-, 2015- and 2019-fold higher at 24, 48, 72, 96 and 120 h compared with the control (76.54; 195.16; 670.91; 1085.71 and 1283.28 U mgP^**−**1^ respectively), obtaining significant differences (*P* ≥ 0.05). MNPp activity (Fig. 1C), similar to LIPp, was only and linearly induced with EHCO but it was always obtained lower than LACp or LIPp activities levels with 8.12, 20.82, 60.58, 161.25 and 190.60 U mgP^**−**1^ for 24, 48, 72, 96 and 120 h, respectively, obtaining significant differences respect to the control (*P* ≥ 0.05).

The results indicate the induction of LACp activity in presence of wheat bran or EHCO, and the linear induction of both LIPp and MNPp activities in presence of EHCO. Interestingly, *P. palmarum* BM-04 is able to overproduce high amounts of LACp with wheat bran or LIPp with EHCO when used as sole carbon and energy source respectively. Due to its high versatility for biotechnological applications, lower requirements and its involvement in petroleum asphaltenes mineralization, LACp activity and non-toxic wheat bran (as inducer) were chosen to obtain a cell-free laccase-enriched OE ultrafiltrate for the next experiments of EHCO biotransformations.

### Oxidative exoenzymes of *P. palmarum* BM-04 catalyse the biotransformation of maltenes, asphaltenes and petroporphyrins-rich fraction of biotreated EHCO

**Figure 2:**
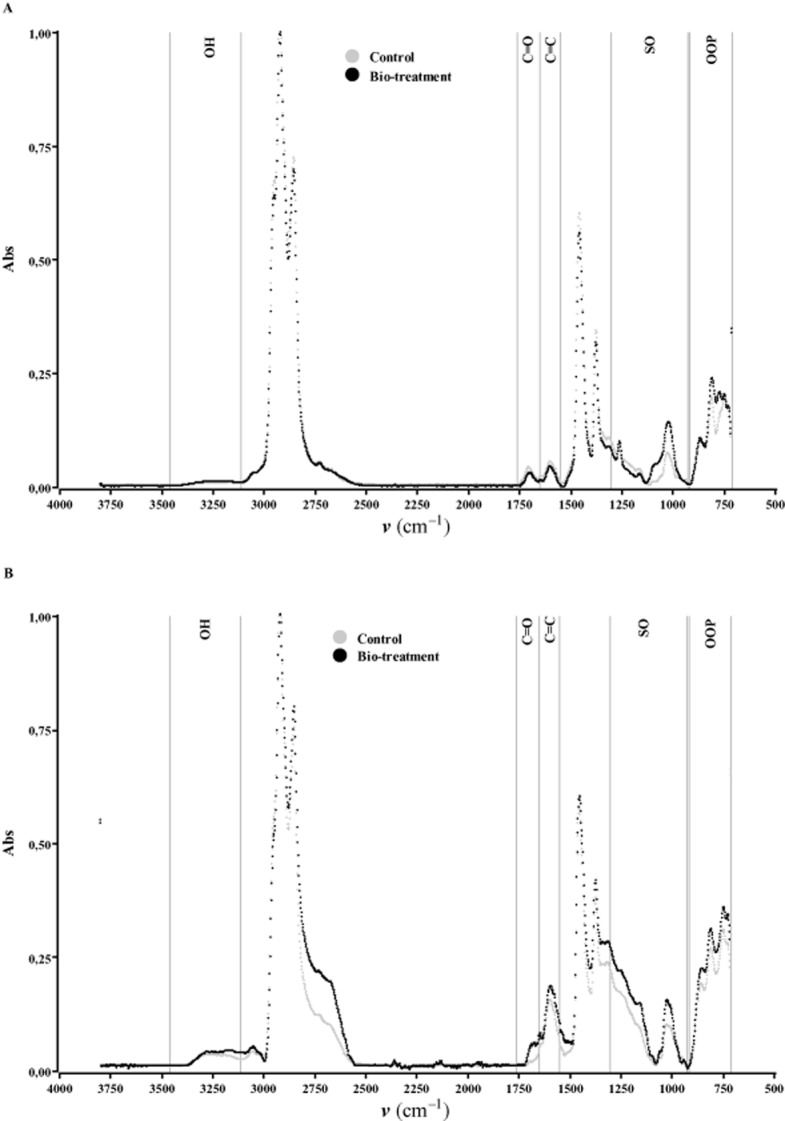
Comparison of normalized second derivative ATR-FT-IR spectra of maltenes (A) and asphaltenes (B) fractions from biotreated EHCO and non-biotreated EHCO (control). Significant differences (*P* ≤ 0.05) among biotreatment and control are indicated for the OH (3460–3110 cm^**−**1^); C=O (1760–1650 cm^**−**1^); C=C (1651–1550 cm^**−**1^); SO (1303–930 cm^**−**1^); and OOP (915–707 cm^**−**1^) regions.

In order to determine the capability of OE to biotransform EHCO, *P. palmarum* BM-04 was grown using wheat bran as sole carbon source to overproduce LACp activity. Figure 2 shows normalized second derivative ATR-FT-IR spectra of maltenes (Fig. 2A) and asphaltenes (Fig. 2B) fractions of biotreated and non-biotreated EHCO. Likewise, it shows the regions where significant differences among biotreatment and control were obtained (*P* ≤ 0.05) using the DGC multiple comparison test, such as: (i) the hydroxyl group (OH: 3110–3460 cm^**−**1^), (ii) the carbonyl group (C=O: 1650–1760 cm^**−**1^), (iii) the carbon-carbon double bond group (C=C: 1550–1650 cm^**−**1^); the sulfoxide group (SO: 930–1303 cm^**−**1^) and (iv) the open of plane group (OOP: 710–915 cm^**−**1^).

ATR-FT-IR spectra in the mid-infrared domain (4000–400 cm^**−**1^) of asphaltenes fractions (Fig. 2B) showed a band around 2900 and 2730 cm^**−**1^ assigned to the C–H stretching vibration of methyl (CH_3_) and methylene (CH_2_) groups. The asymmetric and symmetric CH(–CH_2_–) stretching vibration absorbing at 2920 cm^−1^ and 2850 cm^−1^, respectively, provides a strongly overlapped bands. The stretching vibration of carbonyl (C=O) groups of the ketones, esthers band can be appreciated at 1595 cm^**−**1^. In the region of 1454 and 1373 cm^**−**1^, it is possible to observe the bands of CH_2_ deformation vibration. The band around 1149 cm^**−**1^ was assigned to the stretching vibration =C–H in the plane. The vibration of the sulfoxide (S=O) group is observed around 1028 cm^**−**1^ conjugated with the aromatic vibration in the plane and the deformation of the stretching vibration C–O characteristic of Ar-O-CH_2_-O-Ar. Moreover, two more peaks at 809 and 858 cm^**−**1^ related with aromatic C–H out-of-plane deformation of a single adjacent hydrogen atom and an aromatic C–H out-of-plane deformation of four adjacent hydrogen atoms. Finally, it is observed a peak near 746 cm^**−**1^ that corresponds to alkyl chains longer than 4 methylene units and the CH_2_ rocking modes of saturated chains. In the case of asphaltenes fraction, it is possible to observe the characteristic bands of polycyclic compounds with aliphatic and alicyclic structures, indicating that it is likely that this fraction is composed of polycyclic aromatic rings with aliphatic substitution.

On the other hand, the variation of the asphaltenes fraction was studied by UV-visible spectrophotometry. The asphaltenes fraction (Asf.27) of the biotreated EHCO showed a reduction in the absorbance of the Soret band, probably, due to the porphyrin degradation catalysed by OE of *P. palmarum* BM-04. This result is correlated with those obtained through ATR-FT-IR analysis (García-Arellano *et al*., 2004).

### *P. palmarum* BM-04 is able to grow under high concentrations of salinity and EHCO contamination

**Figure 3:**
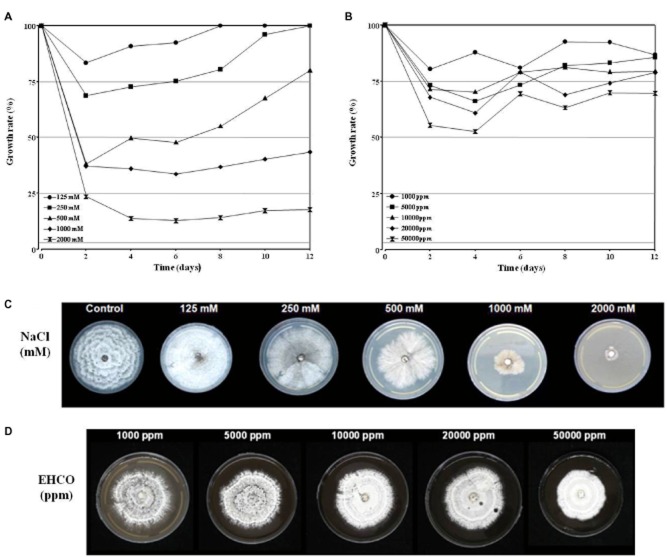
Growth rate of *P. palmarum* BM-04 in BSM medium with different concentrations of NaCl (A, C) and EHCO (B, D).

In order to determine the potential of *P. palmarum* BM-04 to grow and tolerate extreme environmental conditions of salinity and EHCO contamination in hypothetical bioremediation processes, the fungus was grown in agar BSM medium with different concentrations of Carabobo-EHCO or NaCl as described in *Experimental procedures*. The DGC multiple comparison tests was used to detected significant differences among treatment and control. Figure 3A and B shows the range of growth in 0, 125, 250, 500, 1000 and 2000 mM of NaCl; and 1000, 5000, 10 000, 20 000 and 50 000 p.p.m. of EHCO respectively.

DGC multiple comparison tests show higher significant differences among the NaCl concentrations in every considered time intervals (*P* ≤ 0.01). Figure 3A shows that there are three main evident groups of growth rate: the first one of high growth (rate ≥ 72%), formed by the concentrations of 125 mM and 250 mM; the second one of intermediate growth, formed by the concentration of 500 mM; and the third one of low growth, formed by the concentrations of 1000 mM and 2000 mM.

**Figure 4:**
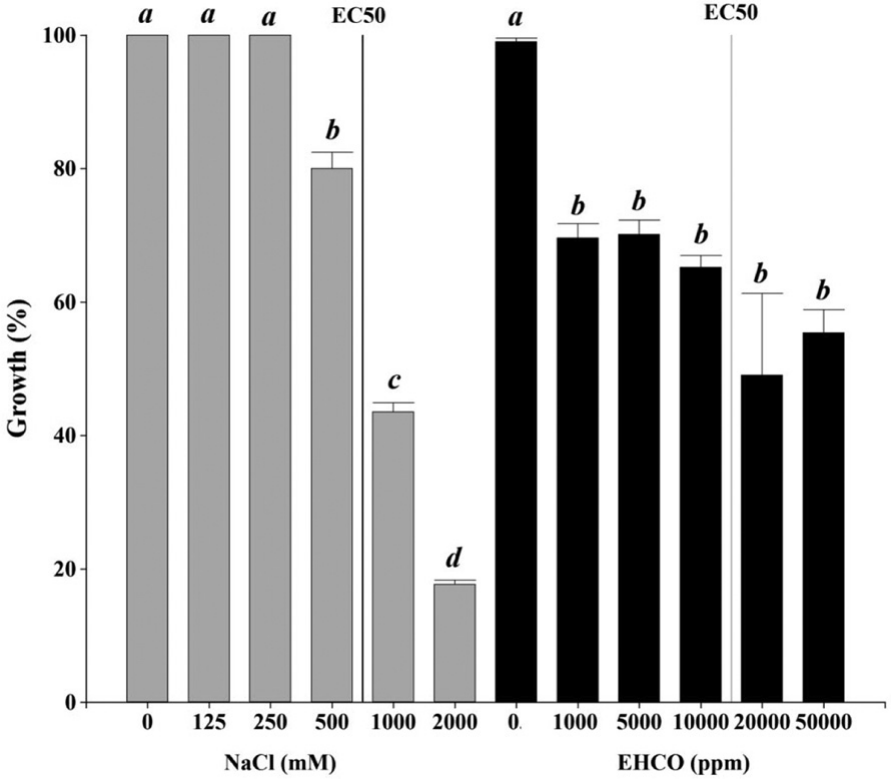
Growth percentage of *P. palmarum* BM-04 in BSM medium with different concentrations of NaCl (grey colour) and EHCO (black colour). Half maximal effective concentration to reduce the 50% of growth (EC50) is showed. Dissimilar letters show significant differences (*P* ≤ 0.05).

The salinity tolerance assays showed the ability of this fungus to grow up to 2000 mM of NaCl, which define this fungus as halotolerant. NOEC and LOEC were 250 mM and 500 mM, respectively, with a decrease in the rate of growth of 20% for 500 mM, about 60% for 1000 mM and 82% for 2000 mM; showing a value of EC50 close to 580 mM of NaCl (Fig. 4).

Figure 3B shows that there are higher significant differences among the EHCO concentrations (*P* ≤ 0.01); the interaction of EHCO levels and time is not significant. Similar to the NaCl behaviour, three main groups of growth rate were detectable: the first one of high growth, formed by the concentration of 1000 p.p.m. of EHCO, with a rate higher than 88%; the second one of intermediate growth, formed by the concentrations of 5000, 10 000 and 20 000 p.p.m. of EHCO, with rates between 75% and 80%; and the third one of low growth, formed by the concentration of 50 000 p.p.m. of EHCO.

The tolerance assays to EHCO showed the ability of this fungus to grow up to 50 000 p.p.m. with a LOEC of 1000 p.p.m. with a decrease in the growth rate of 33%; showing an EC50 of 16 300 p.p.m. (Fig. 4). Nevertheless, non-significant differences of inhibition or normal growth rate to 50 000 p.p.m. were obtained. Also, another study (data no show) revealed the ability of this fungus to grow in a wide range of pH (4–10). These results define *P. palmarum* BM-04 as an alkaliphilic, EHCO-tolerant and halotolerant fungus.

## Discussion

Fungal oxidative exoenzymes lacking substrate specificity play an essential role in the cycling of soil organic matter (Luis *et al*., 2004), and have a potential role in the transformation of contaminant and xenobiotics compounds (for reviews see Pernía *et al*., 1988). Furthermore, it is recognized that the transformation of polymeric substances into partially degraded soluble and oxidized products, can lead to increased availability by microorganisms (Gianfreda and Rao, 2004). Considering the high aromaticity and recalcitrancy levels of EHCO from the OOB, this work was focused on the first step in the transformation of insoluble compounds, which is usually catalysed by oxidative extracellular enzymes (Foght, 2004).

In this work it has been shown that *P. palmarum* BM-04 is able to overproduce mainly LACp when grown using wheat bran or LIPp when grown using EHCO as sole carbon and energy source (Fig. 1). Besides, although the LACp activity was strongly induced with wheat bran, also was weakly induced with EHCO, suggesting a relationship between the LDS and the EHCO biotransformation in *P. palmarum* BM-04. LIPp is able to directly oxidize non-phenolic units; MNPp and LACp to oxidize preferably phenolic units, but they also act on non-phenolic units when mediators, such as ABTS, are present in the reaction mixture (Martínez *et al*., 1996; 2005; Ruiz-Dueñas *et al*., 1999; Saparrat *et al*., 2002; Gianfreda and Rao, 2004). For this reason, the use of laccase-mediator systems is a promising alternative for biotechnological processes with environmental applications (Dávila and Vázquez-Duhalt, 2006).

In the reaction mixture of EHCO biotransformation experiments, 2,2′-azino-bis(3-ethylbenzothiazoline-6-sulfonic acid (ABTS) was used as a mediator to lead the laccase-induced oxidation of phenolic as non-phenolic units. The strongly induced LACp activity with wheat bran could also be due to the presence of inducers such as phenolic, polyphenolic and other aromatic compounds related to lignin (Salas *et al*., 1995; Gianfreda *et al*., 1999). The laccases catalyses the removal of an electron and a proton from phenolic hydroxyl or aromatic amine groups to form free radicals, and constitute an important step in the initial transformation of lignin (Dávila and Vázquez-Duhalt, 2006). Interestingly, Chen and colleagues (2011) reported the laccase production of *Pestalotiopsis* sp. J63 with various lignocellulosic substrates obtaining high levels of activity using rice straw powder (10 700 IU g^**−**1^) and sugarcane bagasse (2000 IU ml^**−**1^).

The significant increase of LIPp activity with EHCO could be due to its polymeric nature that comprises a diverse collection of aromatics and PAHs compounds, typical components of high asphaltenes content petroleum (León and Kumar, 2005). Similar result of extracellular ligninolytic peroxidases over-induction was obtained with *Fusarium solani* HP-1 growing in minimal medium with EHCO as sole carbon source (Naranjo *et al*., 2007).

Asphaltenes is recognized as the most recalcitrant fraction of crude oil because of its high degree of aromaticity. Enzymatic oxidation of PAHs, resins and asphaltenes molecules can lead to an improved availability by microorganisms. Statistical analysis of ATR-FT-IR spectroscopic data shows that indeed the most significant changes occurs in the region of SO and vibration bands of OOP groups. The changes in the SO group region characteristic band at 1028 cm^**−**1^, and in the region around 710–915 cm^**−**1^ characteristic of the vibration OOP of aromatic rings (Fig. 2A and B), suggest that they are linked to an effect of EHCO oxidation catalysed by OE of *P. palmarum* BM-04. Ayala and colleagues (2012) described that the CPO from *C. fumago* is able to catalyse the conversion of organosulfur compounds to sulfoxides and sulfones.

**Figure 5:**
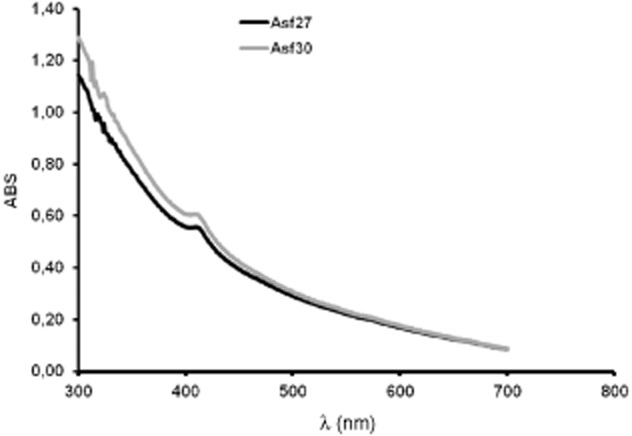
UV visible absorption spectra of asphaltenes fraction from biotreated EHCO (Asf.27) and non-biotreated EHCO (Asf.30).

Besides, fraction of asphaltenes from biotreated EHCO (Fig. 5) shows a band centred on 410 nm due to the presence of vanadylpetroporphyrins indicating that the biotreatment applied to the EHCO can induce a petroporphyrins oxidation. This phenomenon could be confirmed through the observed variations in the infrared spectra, specifically at sulfoxides and carbonyls regions associated with enzymatic oxidation (García-Arellano *et al*., 2004). This result confirms those obtained through analysis by ATR-FT-IR.

On the other hand, in this work it has been shown that *P. palmarum* BM-04 is able to grow under extreme conditions of salinity and high levels of EHCO (Fig. 3 and 4), through the colony growth rate evaluation on selective media test widely used to determine toxicity and capabilities of microorganisms to degrade hydrocarbons (Davis and Westlake, 1979; April *et al*., 1998; Meysami and Baheri, 2003; Hughes *et al*., 2007; Husaini *et al*., 2008). The characteristics of tolerance studied in this work are crucial because they hypothetically reveal the high potential of *P. palmarum* BM-04 to grow in presence of drilling waste impregnated with EHCO and highly salinized water-based drilling fluids, which are mainly constituted by a wide range of corrosive compounds such as sodium bicarbonate, sodium carbonate, potassium chloride, potassium hydroxide, glycol, and sodium hydroxide, thickeners and lubricants, among others, which radically increase the alkalinity and salinity levels of soils and destroy its structure.

As mentioned, oxidative exoenzymes produced by ligninolytic fungi, including peroxidases and laccases, are non-specific biocatalysts with the capacity to produce free radicals. These two characteristics are useful for environmental purposes because they allow oxidizing a wide range of organic compounds with different chemical structures (Dávila and Vázquez-Duhalt, 2006).

Finally, in this article, the OE-overproducing *P. palmarum* BM-04 was identified by formal molecular methods, and is described as a promising hydrocarbonoclastic, alkaliphilic, halotolerant and EHCO-tolerant fungus that could be used efficiently for environmental biocatalysis purposes in extreme environmental conditions of alkalinity, salinity and EHCO contamination, such as drilling waste from the OOB. Our next goal is the cloning and characterization of laccase and lignin peroxidase encoding genes of *P. palmarum* BM-04, in order to study the catalytic potential and operational stability of these fungal exoenzymes.

## Experimental procedures

### Fungal strain, maintenance and growth

The filamentous fungus *Pestalotiopsis palmarum* BM-04 (Cooke) Steyaert 1949 was isolated from the Guanoco Natural Asphalt Lake, Sucre State, Venezuela (Naranjo *et al*., 2007). The fungus was cultivated in Power medium (Pw) to induce sporulation and Basic Salt Medium was used to determine the optimal conditions of growth (Naranjo *et al*., 2004). The fungus is preserved in the Culture Collection of Energy and Environmental Area at the IDEA under deposit number BM-04, and in the Venezuelan Center of Culture Collections (CVCM-UCV) under deposit number 1787.

### Molecular and taxonomic identification of *P. palmarum* BM-04

Genomic DNA isolation was performed following the protocol described by Naranjo and colleagues (2007). For molecular identification, PCR amplifications of ITS region were performed using 0.125 μM of the following specific primers: ITS1F (forward): 5′-CTTGGTCATTTAGAGGAAGTAA-3′ and ITS4R (reverse): 5′-TCCTCCGCTTATTGATATGC-3′. PCR reactions were performed in an Eppendorf Mastercycler *ep*gradient S using a final concentration of 0.20 mM of each deoxyribonucleotide triphosphate, 1.5 mM of MgCl_2_ and 50–70 ng of genomic DNA as template. PCR conditions consisted of an initial denaturation at 95°C for 5 min, and 30 cycles of amplification at 95°C for 1 min, annealing at 56°C for 1 min, extension at 72°C for 1 min, and final extension at 72°C for 7 min. The DNA fragment obtained was sequenced using an ABI Prism™ 310 Genetic Analizer (Applied Biosystems, Foster City, Calif.). All other nucleic acid manipulations were performed by standard methods (Sambrook *et al*., 1989). Nucleotide sequences analysis was performed using the DNA Base Sequence Assembly Software Version 3.5.3.216 (http://www.dnabaser.com), BLASTN (Altschul *et al*., 1997) and FASTA (Pearson and Lipman, 1988).

### Detection of specific oxidative exoenzymes (OE) from the LDS in culture broth

Fresh spores of *P. palmarum* BM-04 were inoculated by triplicate in 250 ml flasks containing 50 ml of modified Cz minimal medium (Naranjo *et al*., 2001) without agar and sucrose (Cx) supplemented with different sole carbon sources as following: (i) Cx with 1% of wheat bran (w/v) doubled autoclaved used as lignocellulolytic substrate, (ii) Cx with 1% of Carabobo-EHCO emulsion (95:5 o/w) and (iii) Cx with sucrose, used as control. Carabobo-EHCO emulsion is water in crude oil emulsion prepared with 95% of EHCO from Carabobo production block of OOB. 2500 p.p.m. of ethoxylated triethyl alcohol was used as emulsifier. The flasks were randomly located in an orbital shaker and incubated at 30°C and 250 r.p.m. One millilitre of supernatant was taken at 24, 48, 72, 96 and 120 h after inoculation, centrifuged to obtain cell-free samples and, used to measure specific enzymatic activities as following:

*LACp activity* (EC 1.10.3.2) in culture broths was measured by colorimetric assay through oxidation of 2,2′-azino-bis(3-ethylbenzothiazoline-6-sulfonic acid (ABTS). The reaction mixture contained 40 μl ABTS (25 mM), 100 μl of sample and 0.1 M sodium tartrate buffer at pH 5 in a final volume of 1000 μl. The ABTS oxidation was monitored during 0–200 s at 436 nm of absorbance and room temperature. The unit of enzymatic activity (U) was defined as 1 mM of ABTS oxidized per ml of supernatant in a minute (ε436 = 29.3 mM^**−**1^ cm^**−**1^) (Saparrat *et al*., 2002).
*LIPp activity* (EC 1.11.1.14) was measured by oxidation of veratryl alcohol in a reaction mixture constituted of 250 μl of veratryl alcohol (20 mM), 40 μl of 1,2% peroxide hydrogen solution, 100 μl of sample and 0.5 M sodium phosphate at pH 3 in a final volume of 1000 μl. Veratryl alcohol oxidation was monitored during 0–200 s at 310 nm of absorbance. The unit of enzymatic activity (U) was defined as 1 μM of veratryl alcohol oxidized per ml of supernatant in a minute (ε310 = 9.3 mM^**−**1^ cm^**−**1^) (Troller *et al*., 1988).
*MNPp activity* (EC 1.11.1.13) was measured by oxidation of phenol red in a reaction mixture constituted of 100 μl of phenol red (0.01%), 100 μl of manganese sulfate cofactor (1 mM), 100 μl of sample and 0.5 M sodium phosphate at pH 5 in a final volume of 1000 μl. Phenol red oxidation was monitored during 0–200 s at 610 nm of absorbance. The unit of enzymatic activity (U) was defined as 1 μM of phenol red oxidized per ml of supernatant in a minute (ε610 = 22.0 mM^**−**1^ cm^**−**1^) (Papinutti *et al*., 2003). All enzymatic activities were perform by triplicate using a Perkin-Elmer Lambda 35 UV/VIS spectrophotometer. Total protein concentration was determined by the Bradford assay (Pierce).

### EHCO biotransformation assay

#### Obtaining a cell-free laccase-enriched OE ultrafiltrate from *P. palmarum* BM-04

Fresh spores of *P. palmarum* BM-04 were inoculated in quadruplicate in 1000 ml flasks containing 400 ml of Cx medium with 1% of wheat bran (w/v) doubly autoclaved as sole carbon source and, incubated at 30°C and 250 r.p.m. for 72 h in order to over induce the LACp activity. The culture broths were collected, mixed and filtered through Nytal filters to separate the mycelium of fungus from the supernatant (Naranjo *et al*., 2007). A tangential microfiltration membrane of 0.45 μm (Millipore, Bedford MA, USA) was used to remove fungal microstructures remaining in the supernatant obtained (1600 ml). The cell-free supernatant was concentrated to 1/10 in 200 ml of sterilized McIlvaine buffer (a mixture of 0.1 molar citric acid and 0.2 molar disodium phosphate, pH 7.0) by a tangential flow ultrafiltration membrane of 10 kDa (Millipore, Bedford MA, USA). The tangential micro/ultra filtration flow was performed using a peristaltic pump Masterflex, Easy-load I/P model XX80EL000, silicone tubing Masterflex 96400-73 and a cassette holder 88 cm^2^ and 0.11 m^2^ Cat.# XX42PMINI (Millipore, USA). The ultra filtration process was performed under a pressure of 8 psi.

### Establishing EHCO biotransformation assay

Fifty millilitres of cell-free laccase-enriched OE ultrafiltrate were added by triplicate in a 250 ml flask containing 50 g of Carabobo-EHCO emulsion (95:5 o/w) and incubated at 30°C and 250 r.p.m. Fifty millilitres of McIlvaine buffer were used as control replacing the OE ultra-filtrate. In both cases 3 mM of ABTS were added as mediator. The enzymatic conversion assay was stopped 20 h after starting the experiment, the emulsion was broken adding 100 ml ethanol, heated and stirred still overnight at 50°C, and the EHCO was dehydrated for the physicochemical characterization.

### Physicochemical characterization of maltenes, asphaltenes and petroporphyrins-rich fractions

#### Separation of maltenes (SAR) and asphaltenes fractions

Asphaltenes and maltenes fractions were separated from crude oil according IP-143 standard method as following: 1 g of EHCO-emulsion 95% (o/w) was suspended in 50 ml of heptane, the mixture was agitated for 2 h at 50°C and then left to settle for additional 24 h at room temperature. The precipitate (asphaltenes) was filtered through filter paper, washed several times with hot n-heptane (65°C) until the solvent became colourless, and then dried for 1 h. Asphaltenes were obtained from filter membrane and dried until constant weight. Heptane was removed from filtrates containing maltenes using a soxhlet equipment keeping concentrate maltenes solution.

### ATR-FT-IR analysis

A Shimadzu Prestige21 spectrometer equipped with a DLATGS detector of high sensitivity, a ceramic light source and a KBr/Germanium beam splitter were employed for spectral measurements. An Attenuated Total Reflectance (ATR) cell was used with a cap in order to avoid volatility of oils. The samples were analysed by means of a diamond-crystal prism manufactured by one bounce on the upper surface. The crystal geometry was a triangle with mirrored 45° angle faces. Air was taken as reference before the collection of each sample spectrum. Data acquisition, made with an absorbance scale, was done from 4000 to 400 cm^−1^ with a 4 cm^−1^ nominal resolution and 100 co-added scans. Five spectra were recorded for each sample deposited on an ATR cell without any preparation or dilution. Three samples from both enzymatic conversion treatment and control were analysed.

### UV-visible analysis

The enzymatic oxidation of petroporphyrins from the asphaltenes fraction was studied using a UV-visible spectrophotometer Shimadzu 3600 according to the manufacture protocol. One milligram of the asphaltene fraction was diluted in 100 ml of toluene and UV-visible spectrum between 300 and 700 nm was recorded. A band centred on 410 nm (Soret band) in the asphaltenes fraction mainly due to the presence of vanadylpetroporphyrins. The loss of the Soret band suggests the disruption of the porphyrin ring via oxidation (García-Arellano *et al*., 2004).

### Tolerance of *P. palmarum* BM-04 to growth in different concentrations of salinity and EHCO

The fungus was cultivated in BSM medium with 0; 1000; 5000; 10 000; 20 000 and 50 000 p.p.m. of Carabobo-EHCO emulsion (50:50 o/w) and 0; 125; 250; 500; 1000 and 2000 mM of NaCl to determine, respectively, the optimal range of growth and tolerance to EHCO or salinity concentration. In both cases, the plates were incubated by triplicate at 30°C for 12 days and, the diameters of colonies were measured every 48 h. The rate of growth was obtained by calculating the diameter of the fungal mycelium in time.

### Statistical analysis

The DGC multiple comparison test was used to detect differences among substrates/conditions for enzymatic activities and fungal growth (Di Rienzo *et al*., 2002). The following toxicological parameters were determined: (i) the NOEC (Non-Observed Effect Concentration), which is the maximum concentration of NaCl or EHCO that produces no statistically significant harmful effects on the growth of fungi compared with controls, and (ii) the LOEC (Lowest-Observed Effect Concentration), which is the lowest concentration that has a statistically significant deleterious effect. Probit analysis was used to determine half maximal effective concentration to reduce the 50% of growth (EC50). All the analyses were made using the InfoStat ver. 2011 (Di Rienzo *et al*., 2010).

## References

[b1] Altschul SF, Madden TL, Schaffer AA, Zhang J, Zhang Z, Miller W, Lipman DJ (1997). Gapped BLAST and PSI-BLAST: a new generation of protein database search programs. Nucleic Acids Res.

[b2] April TM, Abbott SP, Foght JM, Currah RS (1998). Degradation of hydrocarbons in crude oil by the Ascomycete *Pseudallescheria boydii* (Microascaceae). Can J Microbiol.

[b3] Ayala M, Hernández-López EL, Perezgasga L, Vázquez-Duhalt R (2012). Reduced coke formation and aromaticity due to chloroperoxidase-catalyzed transformation of asphaltenes from Maya crude oil. Fuel.

[b4] Chaillan F, Fleche A, Bury E, Phantavong Y, Grimont P, Saliot A, Oudot J (2004). Identification and biodegradation potential of tropical aerobic hydrocarbon-degrading microorganisms. Res Microbiol.

[b5] Chen HY, Xue DS, Feng XY, Yao SJ (2011). Screening and production of ligninolytic enzyme by a marine-derived fungal *Pestalotiopsis* sp. J63. Appl Biochem Biotechnol.

[b6] Dávila A, Vázquez-Duhalt R (2006). Enzimas ligninolíticas fúngicas para fines ambientales. Mensaje Bioquímico.

[b7] Davis JS, Westlake DWS (1979). Crude oil utilization by fungi. J Microbiol.

[b9] Di Rienzo JA, Guzmán AW, Casanoves F (2002). A multiple comparisons method based on the distribution of the root node distance of a binary tree. J Agric Biol Environ Stat.

[b8] Di Rienzo JA, Casanoves F, Balzarini MG, González L, Tablada M, Robledo CW (2010). InfoStat Versión 2010.

[b10] Fedorak PM, Semple KM, Vázquez-Duhalt R, Westlake DWS (1993). Chloroperoxidase mediated modifications of petroporphyrins and asphaltenes. Enzyme Microb Technol.

[b11] Fish RH, Komlenic JJ, Wines BK (1984). Characterization and comparison of vanadyl and nickel compounds in heavy crude petroleums and asphaltenes by reverse-phase and size-exclusion liquid chromatography/graphite furnace atomic absorption. Anal Chem.

[b12] Foght JM, Vázquez-Duhalt R, Quintero-Ramirez R (2004). Whole-cell bio-processing of aromatic compounds in crude oil and fuels. Petroleum Biotechnology: Developments and Perspectives.

[b13] García-Arellano H, Buenrostro-Gonzalez E, Vázquez-Duhalt R (2004). Biocatalytic transformation of petroporphyrins by chemical modified cytochrome c. Biotechnol Bioeng.

[b14] Gianfreda L, Rao M (2004). Potential of extracellular enzymes in remediation of polluted soil: a review. Enzyme Microb Technol.

[b15] Gianfreda L, Xu F, Bollag JM (1999). Laccases: a useful group of oxidoreductive enzymes. Bioremed J.

[b16] Hao JJ, Tian XJ, Song FQ, He XB, Zhang ZJ, Zhang P (2006). Involvement of lignocellulolytic enzymes in the decomposition of leaf litter in a subtropical forest. J Eukaryot Microbiol.

[b17] Hughes KA, Bridge P, Clark MS (2007). Tolerance of Antarctic soil fungi to hydrocarbons. Sci Total Environ.

[b18] Husaini A, Roslan HA, Hii KSY, Ang CH (2008). Biodegradation of aliphatic hydrocarbon by indigenous fungi isolated from used motor oil contaminated sites. World J Microbiol Biotechnol.

[b19] Kim MJ, Lee H, Choi YS, Kim GH, Huh NY, Lee S (2010). Diversity of fungi in creosote-treated crosstie wastes and their resistance to polycyclic aromatic hydrocarbons. Antonie Van Leeuwenhoek.

[b20] Kunamneni A, Ballesteros A, Plou FJ, Alcalde M, Méndez-Vilas A (2007). Fungal laccase, a versatile enzyme for biotechnological applications. Communicating Current Research and Educational Topics and Trends in Applied Microbiology.

[b21] León V, Kumar M (2005). Biological upgrading of heavy crude oil. Biotechnol Bioprocess Eng.

[b22] León Y, De Sisto A, Inojosa Y, Malaver N, Naranjo-Briceño L (2009). Identificación de biocatalizadores potenciales para la remediación de desechos petrolizados de la Faja Petrolífera del Orinoco (2009). RET.

[b23] Luis P, Walthera G, Kellnera H, Martinb F, Buscot F (2004). Diversity of laccase genes from basidiomycetes in a forest soil. Soil Biol Biochem.

[b37] Maharachchikumbura SSN, Guo L-D, Chukeatirote E, Bahkali AH, Hyde KD (2011). Pestalotiopsis – morphology, phylogeny, biochemistry and diversity. Fungal Divers.

[b24] Martínez AT, Speranza M, Ruiz-Dueñas FJ, Ferreira P, Camarero S, Guillén F (2005). Biodegradation of lignocellulosics: microbial, chemical, and enzymatic aspects of the fungal attack of lignin. Int Microbiol.

[b25] Martínez MJ, Ruiz-Dueñas FJ, Guillén F, Martínez AT (1996). Purification and catalytic properties of two manganese-peroxidase isoenzymes from *Pleurotus eryngii*. Eur J Biochem.

[b26] Meysami P, Baheri H (2003). Pre-screening of fungi and bulking agents for contaminated soil bioremediation. Adv Environ Res.

[b29] Naranjo L, Martín de Valmaseda E, Bañuelos O, López P, Riaño J, Casqueiro J, Martín JF (2001). The conversion of pipecolic acid into lysine in *Penicillium chrysogenum* requires pipecolate oxidase and saccharopine reductase: characterization of the *lys*7 gene encoding saccharopine reductase. J Bacteriol.

[b28] Naranjo L, Martín de Valmaseda E, Casqueiro J, Ullán RV, Lamas M, Bañuelos O, Martín JF (2004). Inactivation of the *lys*7 gene, encoding saccharopine reductase in *Penicillium chrysogenum*, leads to accumulation of the secondary metabolite precursors piperideine-6-carboxylic acid and pipecolic acid from α-aminoadipic acid. Appl Environ Microbiol.

[b27] Naranjo L, Urbina H, De Sisto A, León V (2007). Isolation of autochthonous non-white rot fungi with potential for enzymatic upgrading of Venezuelan extra-heavy crude oil. Biocatal Biotransformation.

[b30] Otterbein L, Record E, Longhi S, Asther M, Moukha S (2000). Molecular cloning of the cDNA encoding laccase from *Pycnoporus cinnabarinus* I-937 and expression in *Pichia pastoris*. Eur J Biochem.

[b31] Oudot JP, Dupont J, Haloui S, Roquebert MF (1993). Biodegradation potential of hydrocarbon-degrading fungi in tropical soil. Soil Biol Biochem.

[b32] Papinutti V, Diorio L, Forchiassin F (2003). Degradación de madera de álamo por *Fomes sclerodermeus*; producción de enzimas ligninolíticas en aserrín de álamo y cedro. Rev Iberoam Micol.

[b33] Pearson W, Lipman D (1988). Improved tools for biological sequence comparison. Proc Natl Acad Sci U S A.

[b34] Pernía B, Demey JR, Inojosa Y, Naranjo-Briceño L (2012). Biodiversidad y potencial hidrocarbonoclástico de hongos aislados de crudo y sus derivados: un meta-análisis. Rev Latinoam Biotecnol Amb Algal.

[b35] Ruiz-Dueñas FJ, Martínez MJ, Martínez AT (1999). Molecular characterization of a novel peroxidase isolated from the lignolytic fungus *Pleurotus eryngii*. Mol Microbiol.

[b36] Russell JR, Huang J, Anand P, Kucera K, Sandoval AG, Dantzler KW (2011). Biodegradation of polyester polyurethane by endophytic fungi. Appl Environ Microbiol.

[b38] Salas C, Lobos S, Larrain J, Salas L, Cullen D, Vicuna R (1995). Properties of laccase isoenzymes produced by the basidiomycete *Ceriporiopsis subvermispora*. Biotechnol Appl Biochem.

[b39] Sambrook J, Fritsch EF, Maniatis T (1989). Molecular Cloning: A Laboratory Manual.

[b41] Saparrat MC, Hammer E (2006). Decolorization of synthetic dyes by the deuteromycete *Pestalotiopsis guepinii* CLPS no. 786 strain. J Basic Microbiol.

[b40] Saparrat MC, Guillen F, Arambarri A, Martínez A, Martínez M (2002). Induction, isolation, and characterization of two laccases from the with rot basidiomycete *Coriolopsis rigida*. Appl Environ Microbiol.

[b42] Strauz OP, Mojelsky TW, Lown EM (1992). The molecular structure of asphaltenes: an unfolding story. Fuel.

[b43] Troller J, Smith J, Leisola M, Kallen J, Winterhalter K, Fiechter A (1988). Crystallization of a lignin peroxidase from the white-rot fungus *Phanerochaete chrysosporium*. Bio Technol.

[b44] Uribe-Álvarez C, Ayala M, Perezgasga L, Naranjo L, Urbina H, Vázquez-Duhalt R (2011). First evidence of mineralization of petroleum asphaltenes by a strain of *Neosartorya fischeri*. Microb Biotechnol.

[b45] Waldo GS, Carlson RMK, Moldowan JM, Peters KE, Penner-Hahn JE (1991). Sulfur speciation in heavy petroleums: information from X-ray absorption near-edge structure. Geochim Cosmochim Acta.

[b46] Yang X-L, Zhang J-Z, Luo D-Q (2012). The taxonomy, biology and chemistry of the fungal Pestalotiopsis genus. Nat Prod Rep.

